# Parenting Practices and Well-Being and Health Behaviors Among Young Asian American Children

**DOI:** 10.1001/jamanetworkopen.2024.54516

**Published:** 2025-01-13

**Authors:** Soyang Kwon, Euisung D. Shin, Tami R. Bartell, Selin Capan

**Affiliations:** 1 Buehler Center for Health Policy and Economics, Feinberg School of Medicine, Northwestern University, Chicago, Illinois; 2 University of Illinois College of Medicine at Chicago, Chicago; 3 Patrick M. Magoon Institute for Healthy Communities, Ann & Robert H. Lurie Children’s Hospital of Chicago, Chicago, Illinois

## Abstract

**Importance:**

Literature suggests that well-being and health status differ by generational status among Asian American individuals.

**Objective:**

To compare young children’s well-being and health behaviors and their parents’ parenting practices among families of second-generation Asian American, third- or later-generation Asian American, and third- or later-generation non-Hispanic White children in the US.

**Design, Setting, and Participants:**

For this survey study, secondary data analysis was conducted from September 2, 2023, to June 19, 2024, using data from the 2018 to 2022 National Survey of Children’s Health participants aged 6 months to 5 years. Parents completed a survey about their positive parenting practices and their child’s psychological well-being and health behaviors via mail or online. Multivariable logistic and linear regression analyses were conducted to compare psychological well-being and health behaviors by study groups and positive parenting practices, adjusting for sociodemographic variables.

**Main Outcomes and Measures:**

Positive parenting practices (ie, reading, storytelling or singing, and family meals), psychological well-being (ie, flourishing, social-emotional development, and self-regulation), and health behaviors.

**Results:**

The analysis included 42 846 participants (5456 in 2018, 5140 in 2019, 7494 in 2020, 12 817 in 2021, and 11 939 in 2022; 46.6% female and 53.4% male). Participants were categorized into 3 groups: 2881 (6.7%) were second-generation Asian American children aged 0 to 5 years, 570 (1.3%) third- or later-generation Asian American children aged 0 to 5 years, and 39 395 (92.0%) third- or later-generation non-Hispanic White children aged 0 to 5 years. Compared with White children, Asian American children were less likely to flourish (odds ratio [OR], 0.57 [95% CI, 0.52-0.63] for second-generation Asian American; OR, 0.82 [95% CI, 0.65-1.03] for third- or later-generation Asian American), have regular bedtimes (OR, 0.80 [95% CI, 0.69-0.92] for second-generation Asian American; OR, 0.66 [95% CI, 0.49-0.88] for third- or later-generation Asian American), and have moderate screen time (OR, 0.92 [95% CI, 0.84-1.00] for second-generation Asian American; OR, 0.82 [95% CI, 0.69-0.97] for third- or later-generation Asian American). Results for regular reading and storytelling or singing were the lowest among second-generation Asian American children (1430 [49.6%] for reading and 1629 [56.5%] for storytelling or singing), followed by the third- or later-generation Asian American children (376 [66.0%] for reading and 412 [72.3%] for storytelling or singing) and than White children (28 628 [72.7%] for reading and 30 375 [77.1%] for storytelling or singing) (*P* < .001). Results for family meals were similar between second-generation (2356 [81.8%]) and third- or later-generation (467 [81.9%]) Asian American children; however, both were significantly lower than for White children (33 928 [86.1%]) (*P* < .001). All 3 positive parenting practices were positively associated with children’s psychological well-being and health behaviors.

**Conclusions and Relevance:**

This survey study of young children found lower likelihoods of flourishing, regular bedtimes, and moderate screen time among Asian American children compared with non-Hispanic White children in the US. Reading and storytelling or singing parenting practices, which were associated with children’s well-being and health behaviors, were particularly less frequent among parents of second-generation Asian American children than those of third- or later-generation Asian American children. Promotion of these practices among Asian immigrant parents may help improve the psychological well-being and health behaviors of their young Asian American children.

## Key Points

**Question:**

Do young children’s well-being and health behaviors and their parents’ parenting practices differ among Asian American and non-Hispanic White families?

**Findings:**

In this survey study of a nationally representative sample of 42 846 young children in the US. Asian American children had lower likelihoods of flourishing, regular bedtimes, and moderate screen time compared with non-Hispanic White children. These lower likelihoods were associated with less frequent parent-child reading and storytelling or singing parenting practices.

**Meaning:**

Promotion of parent-child reading and storytelling or singing parenting practices among Asian immigrant parents may help improve the psychological well-being and health behaviors of their young Asian American children.

## Teaser

This survey study of Asian American and White parents of children aged 6 months to 5 years examines parenting practices and children’s psychological well-being and health behaviors.

## Introduction

Early childhood is a critical period for cognitive, social, emotional, and physical development that establishes the basis for future health and wellness.^1,2,3^ During this time from birth to 5 years of age, flourishing and social-emotional development are key indicators of psychological well-being.^4,5^ Flourishing represents not only the absence of negative health outcomes but also the presence of protective health-related factors, such as one’s ability to cope with stress and foster positive relationships.^4,6,7^ For young children, the operationalization of flourishing includes affect, resilience, curiosity, and affection.^4,6,8^ Flourishing is associated with lower risks of anxiety, antisocial behaviors, and psychiatric morbidities later in life.^4,9,10,11^ Overlapping with some flourishing indicators, social and emotional development expand on these domains. Often conceptualized together with the growing understanding of their interrelationship,^12^ early social-emotional development refers to a child’s ability to “form close and secure adult and peer relationships; experience, regulate, and express emotions in socially and culturally appropriate ways; and explore the environment and learn—all in the context of family, community, and culture.”^13^ Within this definition, self-regulation becomes a foundational social-emotional skill.^12^ Healthy social-emotional development is associated with future academic achievement,^12^ social competence with peers,^14^ and reduced juvenile delinquency,^15^ while greater childhood self-regulation is associated with better physical health and financial outcomes as well as decreased criminality in adulthood.^16^

Asian American individuals are among the fastest growing US racial and ethnic groups, with their population projected to surpass 46 million by 2060.^17,18^ Many Asian American youths experience mental health challenges, including social anxiety, low self-esteem, and depressed mood.^19,20^ Furthermore, literature suggests that well-being status differs by generational status among Asian American adults.^21^ However, less is known about the early childhood well-being of Asian American populations, specifically regarding how generational status affects psychological well-being and health behaviors among young Asian American children and how the parenting practices of their caregivers might affect these associations. Given the lifelong impacts of early childhood development, understanding these formative dynamics is key to evaluating the needs of these children and then forming targeted policies and programs that can most benefit Asian American children.

To address this knowledge gap, this study aimed to compare the psychological well-being (ie, flourishing, social-emotional development, self-regulation) and health behaviors (ie, regular bedtimes, screen time) of second-generation non-Hispanic Asian American, third- or later-generation non-Hispanic Asian American, and third- or later-generation non-Hispanic White children aged 0 to 5 years. The secondary aim was to compare positive parenting practices among the parents of the study children. We hypothesized that second-generation Asian American children would have the poorest psychological well-being and health behavior outcomes, followed by third- or later-generation Asian American children and then non-Hispanic White children. Additionally, we hypothesized that these group differences would be partly explained by parenting practices.

## Methods

### Study Participants

Secondary data analysis was conducted from September 2, 2023, to June 19, 2024, using the 2018, 2019, 2020, 2021, and 2022 National Survey of Children’s Health (NSCH) data. This survey study reports sample size, margin of sampling error, weighting attributes, the full text of the questions, answer options, the survey mode, and the population under study in accordance with the American Association for Public Opinion Research (AAPOR) reporting guideline. The NSCH was a web- and mail-based national survey of noninstitutionalized US children aged 6 months to 17 years conducted annually from June or July to January. The NSCH randomly sampled addresses from the US Census Bureau’s Master Address file, including 50 states and the District of Columbia, to mail a screening questionnaire. On the basis of screening questionnaire responses, 1 child per household was randomly selected as an NSCH participant. An adult familiar with the child’s health (respondent) then completed a web- and mail-based questionnaire in English or Spanish. Annual response rates ranged from 37% to 42%. The current analysis included participants who identified as second-generation Asian American, third-or later-generation Asian American, or non-Hispanic White and excluded participants whose survey respondent was not a parent (eg, grandparent). Parents provided written informed consent. This study was exempted from ethics review by the institutional review board of the Ann and Robert H. Lurie Children’s Hospital because it used deidentified publicly available data.

### Study Groups

The study groups of interest included second-generation Asian American children aged 0 to 5 years, third-or later-generation Asian American children aged 0 to 5 years, and White children aged 0 to 5 years. A child was categorized as Asian American if a respondent reported the child’s race as Asian alone (from the categories of American Indian or Alaska Native alone, Asian alone, Black or African American alone, Native Hawaiian or other Pacific Islander alone, White alone, or ≥2 races) and the child’s ethnicity as non-Hispanic (from the categories of Hispanic or non-Hispanic). A child was categorized as White if a respondent reported the child’s race as White alone and ethnicity as non-Hispanic. Generational status was determined by the child’s and parents’ birthplaces: a child was categorized as second generation if the child was born in the US and at least 1 parent was born elsewhere and as third- or later-generation if the child and both parents were born in the US.

### Outcome Measures

#### Positive Parenting Practices

Parenting practices were assessed using 3 survey questions to assess the frequency (0 days, 1-3 days, 4-6 days, or every day during the past week) of engagement in positive parenting practices: “During the past week, (1) how many days did you or other family members read to this child?” (reading); (2) “how many days did you or other family members tell stories or sing songs to this child?” (storytelling or singing); and (3) “on how many days did all family members who live in the household eat a meal together?” (family meals).^22^ Responses of 4 to 6 days or every day were considered regular engagement (ie, regular reading, regular storytelling or singing, and regular family meals).

#### Psychological Well-Being

Flourishing was assessed using 4 flourishing survey items: “How often (1) is this child affectionate and tender with you” (affection), (2) “does this child bounce back quickly when things do not go their way” (resilience), (3) “does this child show interest and curiosity in learning new things” (curiosity), and (4) “does this child smile and laugh?” (affect).^6,23^ Each item was rated on a 4-point scale, with 1 indicating never; 2, sometimes; 3, usually; and 4, always. Those who responded always or usually to all 4 questions were categorized as flourishing.^6,23^

Guided by the Healthy and Ready to Learn evaluation,^24^ social-emotional development and self-regulation items were selected. The social-emotional development domain was assessed using responses to 3 questions: (1) “How often can this child explain things they have seen or done so that you know what happened?” (explain things); (2) “how often can this child share toys or games with other children?” (share with others); and (3) “how often does this child show concern when they see others who are hurt or unhappy?” (concern for others). Each item was rated on a 5-point scale, with 1 indicating never; 2, sometimes; 3, about half the time; 4, most of the time; and 5, always. Self-regulation was assessed using responses to 4 questions rated on the same 5-point scale: (1) “How often does this child become angry or anxious when going from one activity to another?” (in the 2018-2021 NSCHs) and “how often does this child have difficulty when asked to end one activity and start a new activity?” (in the 2022 NSCH) (transition); (2) “when excited or all wound up, how often can this child calm down quickly?” (in the 2018-2021 NSCHs) and “how often does this child have trouble calming down?” (in the 2022 NSCH) (calm down); (3) “how often is this child easily distracted?” (distracted); and (4) “how often does this child lose control of his or her temper when things do not go his or her way?” (in the 2018-2021 NSCHs) and “how often does this child lose their temper?” (in the 2022 NSCH) (temper). All self-regulation items, except for the 2018 to 2021 calm down item, were reverse coded such that a higher score indicated higher self-regulation. These questions were only fielded to participants aged 3 to 5 years. A social-emotional score was created by averaging the 3 social-emotional item scores, with a higher score indicating better social-emotional development. Similarly, a self-regulation score was created by averaging the 4 social-emotional item scores, with a higher score indicating higher self-regulation.

#### Health Behaviors

Health behaviors of interest included regular bedtime and moderate screen time. These behaviors were selected based on the World Health Organization’s 24-Hour Movement Guidelines,^25^ which incorporate physical activity, sleep, and sedentary behaviors (including screen time) in a holistic approach to healthy lifestyles in early childhood. Sleep and screen time were assessed individually using a single question from the NSCH. Those with a usually or always response to the sleep question were considered to have a regular bedtime.^26^ Responses of less than 1 hour for children aged 0 to 1 year and less than 1 hour or 1 hour for children aged 2 to 5 years were considered as having moderate screen time.^27^ Physical activity could not be assessed due to lack of NSCH domain questions.

#### Other Variables

Several sociodemographic factors were considered as confounders, similar to prior investigations^6,28^: child’s age and sex; federal poverty level (<100% [below the federal poverty level], 100 to <200%, 200 to <400%, or ≥400%)^29^; respondent’s marital status (married vs nonmarried) and emotional support for parenting (yes or no: “During the past 12 months, was there someone that you could turn to for day-to-day emotional support with parenting or raising children?”); parents’ employment status (employed or not employed); mother’s educational attainment (less than a bachelor’s degree or bachelor’s degree or higher); and neighborhood support (supportive or not supportive).^29^ Primary language spoken at home (English or language other than English) was not considered a confounder due to its association with study group categories.

### Statistical Analysis

Statistical analyses were conducted using SAS software, version 9.4 survey procedures (SAS Institute Inc) without accounting for NSCH weighting design, as sampling was not designed to represent populations by generational status. χ^2^ Tests were used to compare participant characteristics among included and excluded participants. Bivariate analyses (eg, analysis of variance, χ^2^ tests, and 2-sided, unpaired *t* tests) were performed to compare study variables among study groups. A 2-sided *P* < .05 was considered statistically significant.

Multivariate logistic and linear regression analyses were conducted to compare outcome variables by study groups and positive parenting practices, adjusted for sociodemographic confounding factors. For covariates with 10% or more missing data (ie, mothers’ educational level and neighborhood support), a missing value category was created. Other variables with less than 10% missing data were treated using a pairwise deletion method. We repeated these analyses separately by survey year (2018-2019 [combined due to small sample size], 2020, 2021, and 2022) as a sensitivity analysis to examine the consistency of results across survey years. We examined the associations between positive parenting practices and outcome variables within the 2 Asian American samples to ensure that the associations in these groups were not masked by the White group, which comprised more than 90% of the entire sample. To evaluate the impact of the excluded-due-to-missing data, we imputed missing data using multiple imputation by chained equations and conducted regression analyses including imputed data.

## Results

Our analysis included 42 846 participants (5456 in 2018, 5140 in 2019, 7494 in 2020, 12 817 in 2021, and 11 939 in 2022; 46.6% female and 53.4% male), excluding 1471 respondents who were not a parent. Excluded participants had lower household income, had a lower educational level among respondents, and were less likely to flourish compared with included participants.

Among the included participants, 2881 (6.7%) were second-generation Asian American, 570 (1.3%) were third- or later-generation Asian American, and 39 395 (92.0%) were White (Table 1). Among 3451 Asian American children, 28 were first generation. Respondents’ mean (SD) age was 37 (6) years for Asian American parents and 35 (6) years for White parents. For the second-generation Asian American group, median (IQR) time living in the US was 13 (8-20) years for respondents and 11 (7-18) years for the other parents, and median (IQR) age at immigration was 24 (18-28) years for respondents and 25 (20-29) years for the other parents.

**Table 1.** Characteristics of 2018 to 2022 National Survey of Children’s Health Participants Aged 0 to 5 Years by Study Group^a^

Characteristic	No. (%) of participants			*P* value
	Second-generation Asian American (n = 2881)	Third- or later- generation Asian American (n = 570)	Third- or later- generation White American (n = 39 395)	
Sex				
Male	1539 (53.4)	294 (51.6)	20 456 (51.9)	.29
Female	1342 (46.6)	276 (48.4)	18 939 (48.1)	
Age group				
6 mo to 2 y	1171 (40.6)	260 (45.6)	17 313 (44.0)	.002
3 to 5 y	1710 (59.4)	310 (54.4)	22 082 (56.0)	
Federal poverty level				
<100% (Below poverty level)	240 (8.3)	35 (6.2)	2205 (5.6)	<.001
100 to <200%	422 (14.3)	73 (12.8)	5075 (12.9)	
200 to <400%	961 (33.4)	169 (29.6)	14 655 (37.2)	
≥400%	1258 (43.7)	293 (51.4)	17 460 (44.3)	
Respondent’s relationship with the child				
Father	1693 (58.8)	226 (39.6)	11 973 (30.4)	<.001
Mother	1188 (41.2)	344 (60.4)	27 422 (69.6)	
Respondent’s marital status				
Married	2690 (93.4)	474 (83.2)	34 227 (86.9)	<.001
Nonmarried	191 (6.6)	96 (16.8)	5168 (13.1)	
Respondent has emotional support for parenting				
Yes	1552 (53.9)	460 (80.7)	36 155 (91.8)	<.001
No	1329 (46.1)	110 (19.3)	3240 (8.2)	
Father’s educational level				
Less than bachelor’s degree	596 (22.9)	162 (33.2)	15 435 (42.3)	<.001
Bachelor’s degree or higher	2011 (77.1)	326 (66.8)	21 029 (57.7)	
Mother’s educational level				
Less than bachelor’s degree	610 (23.3)	128 (24.4)	12 733 (33.2)	<.001
Bachelor’s degree or higher	2012 (76.7)	396 (75.6)	25 678 (66.8)	
Father’s employment				
Employed	2429 (93.8)	460 (94.3)	34 468 (94.8)	.09
Not employed	161 (6.2)	28 (5.7)	1903 (5.2)	
Mother’s employment				
Employed	1571 (60.4)	412 (79.1)	28 140 (73.5)	<.001
Not employed	1032 (39.6)	109 (20.9)	10 150 (26.5)	
No. of children in household				
1	1180 (41.0)	227 (39.8)	13 280 (33.7)	<.001
2	1353 (47.0)	271 (47.5)	18 298 (46.5)	
≥3	348 (12.1)	72 (12.6)	7817 (19.8)	
Primary language spoken at home				
English	1396 (48.5)	534 (93.7)	39 112 (99.6)	<.001
Language other than English	1484 (51.5)	36 (6.3)	169 (0.43)	
Supportive neighborhood^b^				
Yes	1488 (53.1)	283 (51.3)	24 741 (63.4)	<.001
No	1320 (46.9)	269 (48.7)	14 255 (36.6)	
Parenting practices				
Reading to child ≥4 d/wk^c^^,^^d^^,^^e^				
Yes	1430 (49.6)	376 (66.0)	28 628 (72.7)	<.001
No	1451 (50.4)	194 (34.0)	10 767 (27.3)	
Tell stories or sing songs to child ≥4 d/wk^c^^,^^d^^,^^e^				
Yes	1629 (56.5)	412 (72.3)	30 375 (77.1)	<.001
No	1252 (43.5)	158 (27.7)	9020 (22.9)	
Family meal ≥4 d/wk^d^^,^^e^				
Yes	2356 (81.8)	467 (81.9)	33 928 (86.1)	<.001
No	525 (18.2)	103 (18.1)	5467 (13.8)	

^a^ All characteristics were self-reported. Data were missing for 3287 for father’s educational level, 1289 for mother’s educational level, 3397 for father’s employment, 1432 for mother’s employment, 115 for primary language spoken at home, and 490 for neighborhood support.

^b^ Neighborhood support was assessed using 3 statements: (1) “people in this neighborhood help each other out”; (2) “we watch out for each other's children in this neighborhood”; and (3) “when we encounter difficulties, we know where to go for help in our community.” Items were rated on a 5-point scale, with 1 indicating definitely agree to 5 indicating definitely disagree. Participants’ neighborhoods were considered supportive if the respondent reported definitely agree to 1 item and somewhat agree or definitely agree to the other 2 items.

^c^ For parenting practices, *P* < .001 between the second-generation Asian American and third- or later-generation Asian American groups.

^d^ For parenting practices, *P* < .001 between the second-generation Asian American and third- or later-generation White American groups.

^e^ For parenting practices, *P* < .001 between the third- or later-generation Asian American and third- or later-generation White American groups.

Results for regular reading and storytelling or singing were the lowest among second-generation Asian American children (1430 [49.6%] reading and 1629 [56.5%] storytelling or singing), followed by third- or later-generation Asian American children (376 [66.0%] reading and 412 [72.3%] storytelling or singing) and then White children (28 628 [72.7%] reading and 30 375 [77.1%] storytelling or singing) (*P* < .001) (Table 1). Results for family meals were similar between the second-generation (2356 [81.8%]) and third- or later-generation (467 [81.9%]) Asian American children; however, both were significantly lower than for White children (33 928 [86.1%]) (*P* < .001).

Results for flourishing were the lowest for second-generation Asian American children (2084 [74.3%]), followed by third- or later-generation Asian American children (468 [83.4%]) and then White children (33 942 [87.1%]) (*P* < .001) (Table 2). Of the 4 flourishing items, resilience showed the largest differences across the study groups (2222 [78.5%] in second-generation Asian American children vs 485 [86.5%] in third- or later-generation Asian American children and 35 323 [90.3%] in White children *P* < .001).

**Table 2.** Comparisons of Flourishing, Health Behaviors, Social-Emotional Development, and Self-Regulation in the 2018 to 2022 National Survey of Children’s Health Participants by Study Group^a^

Measure	No. (%) of participants^b^			*P* value
	Second-generation Asian American	Third- or later- generation Asian American	Third- or later- generation White American	
**Participants aged 0-5 y**				
No. of participants	2881	570	39 395	
Flourishing	2084 (74.3)^c^^,^^d^	468 (83.4)^e^	33 942 (87.1)	<.001
Usually or always affectionate and tender (affection)	2672 (94.7)^c^^,^^d^	548 (97.7)	38 103 (97.4)	<.001
Usually or always bounce back quickly when things do not go their way (resilience)	2222 (78.5)^c^^,^^d^	485 (86.5)^e^	35 323 (90.3)	<.001
Usually or always interested and curious in learning new things (curiosity)	2693 (95.2)^d^	539 (96.1)	37 972 (97.1)	<.001
Usually or always smile and laugh (affect)	2809 (98.9)	554 (98.8)	38 779 (99.1)	.30
Health behaviors				
Regular bedtime	2578 (90.4)^d^	507 (90.5)^e^	36 904 (94.3)	<.001
Moderate screen time	1413 (49.8)^d^	279 (49.7)^e^	21 426 (54.8)	<.001
**Participants aged 3-5 y**				
No.	1710	310	22 082	
Social-emotional score, mean (SD)	4.16 (0.73)^c^^,^^d^	4.24 (0.66)	4.25 (0.58)	<.001
Explain things	4.25 (0.96)^d^	4.35 (0.89)	4.34 (0.85)	<.001
Share with others	4.25 (0.88)	4.27 (0.77)	4.25 (0.66)	.78
Concern for others	3.97 (1.07)^d^	4.09 (0.97)	4.17 (0.84)	<.001
Self-regulation score, mean (SD)	3.68 (0.63)^d^	3.69 (0.66)	3.75 (0.57)	<.001
Transition well	3.86 (0.97)^d^	3.96 (0.89)^e^	4.06 (0.08)	<.001
Calm down quickly	3.82 (0.88)^d^	3.70 (0.84)^e^	3.65 (0.83)	<.001
Not easily distracted	3.45 (0.95)^d^	3.50 (0.88)	3.59 (0.81)	<.001
Control temper	3.60 (0.93)^d^	3.62 (0.86)	3.71 (0.77)	<.001

^a^ Data were missing for 530 for flourishing, 278 for bedtime, 316 for screen time, 317 for social-emotional items, and 335 for self-regulation items. A higher score indicates better social-emotional development and higher self-regulation.

^b^ Unless otherwise indicated.

^c^ *P* < .001 between the second-generation Asian American and third- or later-generation Asian American groups.

^d^ *P* < .001 between the second-generation Asian American and third- or later-generation White American groups.

^e^ *P* < .001 between the third- or later-generation Asian American and third- or later-generation White American groups.

Compared with White children, Asian American children had lower proportions of regular bedtimes (2578 [90.4%] in the second-generation group and 507 [90.5%] in the third- or later generation group vs 36 904 [94.3%] in the White group; *P* < .001) and moderate screen time (1413 [49.8%] in the second-generation group and 279 [49.7%] in the third- or later generation group vs 21 426 [54.8%] in the White group; *P* < .001), with no difference between the 2 Asian American groups. Among children aged 3 to 5 years, the second-generation but not the third- or later-generation Asian American group had lower mean (SD) social-emotional (4.16 [0.73] and 4.24 [0.66], respectively) and self-regulation (3.68 [0.63] and 3.69 [0.66], respectively) scores compared with the White group (4.25 [0.58] and 3.75 [0.57], respectively) (*P* < .001).

After adjusting for confounders, regular reading and regular family meals were significantly associated with flourishing (odds ratio [OR], 1.14; 95% CI, 10.6-1.22 and OR, 1.25; 95% CI, 1.15-1.34, respectively), regular bedtimes (OR, 2.10; 95% CI, 1.91-2.31 and OR, 1.55; 95% CI, 1.40-1.71, respectively), and moderate screen time (OR, 1.34; 95% CI, 1.28-1.41 and OR, 1.55; 95% CI, 1.46-1.64, respectively) among children aged 0 to 5 years (*P* < .001) (Table 3). Regular storytelling or singing was associated with flourishing (OR, 1.36; 95% CI, 1.26-1.46) and regular bedtimes (OR, 1.22; 95% CI, 1.11-1.35; *P* < .001). Second-generation and third- or later-generation Asian American children were less likely to have regular bedtimes (OR, 0.80; 95% CI, 0.69-0.92 and OR, 0.66; 95% CI, 0.49-0.88, respectively) and have moderate screen time (OR, 0.92; 95% CI, 0.84-1.00 and OR, 0.82; 95% CI, 0.69-0.97, respectively) compared with White children, whereas second-generation (OR, 0.57; 95% CI, 0.52-0.63) but not third- or later-generation (OR, 0.82; 95% CI, 0.65-1.03) Asian American children were less likely to flourish compared with White children.

**Table 3.** Multivariable Logistic Regression Models for Flourishing, Regular Bedtime, and Moderate Screen Time in the 2018 to 2022 National Survey of Children’s Health Participants Aged 0 to 5 Years^a^

Characteristic	Odds ratio (95% CI)		
	Flourishing (n = 42 316)	Regular bedtime (n = 42 568)	Moderate screen time (n = 42 530)
Age, median (IQR), y	0.83 (0.82-0.85)	0.96 (0.93-0.99)	0.77 (0.76-0.78)
Sex			
Male	0.76 (0.72-0.80)	1.12 (1.03-1.21)	0.96 (0.93-1.00)
Female	1 [Reference]	1 [Reference]	1 [Reference]
Federal poverty level			
<100% (Below poverty level)	0.56 (0.50-0.63)	0.57 (0.49-0.67)	0.88 (0.80-0.97)
100 to <200%	0.76 (0.69-0.84)	0.75 (0.66-0.86)	0.84 (0.79-0.90)
200 to <400%	0.87 (0.81-0.93)	0.85 (0.77-0.94)	0.83 (0.79-0.86)
≥400%	1 [Reference]	1 [Reference]	1 [Reference]
Respondent’s marital status			
Nonmarried	0.99 (0.90-1.07)	0.70 (0.63-0.76)	0.92 (0.87-0.99)
Married	1 [Reference]	1 [Reference]	1 [Reference]
Respondent has emotional support for parenting			
Yes	1.48 (1.36-1.61)	1.38 (1.23-1.55)	1.07 (1.00-1.15)
No	1 [Reference]	1 [Reference]	1 [Reference]
Mother’s educational level			
Missing	1.08 (0.92-1.27)	0.99 (0.79-1.24)	0.69 (0.61-0.78)
Less than bachelor’s degree	0.94 (0.88-1.01)	0.73 (0.67-0.81)	0.60 (0.57-0.63)
Bachelor’s degree or higher	1 [Reference]	1 [Reference]	1 [Reference]
Neighborhood support			
Missing	1.37 (1.02-1.85)	1.29 (0.88-1.88)	1.83 (1.45-2.30)
Yes	1.57 (1.48-1.67)	1.40 (1.29-1.53)	1.28 (1.23-1.33)
No	1 [Reference]	1 [Reference]	1 [Reference]
Read to child ≥4 d/wk			
Yes	1.14 (1.06-1.22)	2.10 (1.91-2.31)	1.34 (1.28-1.41)
No	1 [Reference]	1 [Reference]	1 [Reference]
Tell stories or sing songs to child ≥4 d/wk			
Yes	1.36 (1.26-1.46)	1.22 (1.11-1.35)	1.05 (0.99-1.11)
No	1 [Reference]	1 [Reference]	1 [Reference]
Family meal ≥4 d/wk			
Yes	1.25 (1.15-1.34)	1.55 (1.40-1.71)	1.55 (1.46-1.64)
No	1 [Reference]	1 [Reference]	1 [Reference]
Study group			
Second-generation Asian American	0.57 (0.52-0.63)	0.80 (0.69-0.92)	0.92 (0.84-1.00)
Third- or later-generation Asian American	0.82 (0.65-1.03)	0.66 (0.49-0.88)	0.82 (0.69-0.97)
Third- or later-generation White American	1 [Reference]	1 [Reference]	1 [Reference]

^a^ All characteristics were self-reported.

After adjusting for confounders, regular reading was associated with higher self-regulation scores among children aged 3 to 5 years (mean [SE] score, 0.03 [0.01]; *P* = .002) (Table 4). Regular storytelling or singing and regular family meals were associated with higher social-emotional scores (mean [SE], 0.10 [0.01] and 0.12 [0.01], respectively) and self-regulation scores (mean [SE], 0.05 [0.01] and 0.11 [0.01], respectively) (*P* < .001). The study group variable was not significantly associated with social-emotional or self-regulation scores.

**Table 4.** Multivariable Linear Regression Models for Social-Emotional and Self-Regulation Scores in the 2018-2022 National Survey of Children’s Health Participants Aged 3 to 5 Years^a^

Characteristic	Social-emotional score (n = 23 785)		Self-regulation score (n = 23 767)	
	Coefficient (SE)	*P* value	Coefficient (SE)	*P* value
Intercept	3.47 (0.03)	<.001	3.28 (0.03)	<.001
Age, y	0.12 (0.005)	<.001	0.05 (0.005)	<.001
Sex				
Male	−0.14 (0.01)	<.001	−0.12 (0.01)	<.001
Female	1 [Reference]	NA	1 [Reference]	NA
Federal poverty level				
<100% (Below federal poverty level)	−0.13 (0.02)	<.001	−0.12 (0.02)	<.001
100 to <200%	−0.06 (0.01)	<.001	−0.04 (0.01)	.006
200 to <400%	−0.03 (0.01)	.002	−0.01 (0.01)	.33
≥400%	1 [Reference]	NA	1 [Reference]	NA
Respondent’s marital status				
Nonmarried	0.02 (0.01)	.08	−0.06 (0.01)	<.001
Married	1 [Reference]	NA	1 [Reference]	NA
Respondent has emotional support for parenting				
Yes	0.09 (0.01)	<.001	0.12 (0.01)	<.001
No	1 [Reference]	NA	1 [Reference]	NA
Mother’s educational level				
Missing	0.03 (0.02)	.22	0.04 (0.02)	.04
Less than bachelor’s degree	0.05 (0.01)	<.001	−0.03 (0.01)	.003
Bachelor’s degree or higher	1 [Reference]	NA	1 [Reference]	NA
Neighborhood support				
Missing	0.19 (0.04)	<.001	0.10 (0.04)	.01
Yes	0.18 (0.01)	<.001	0.15 (0.01)	<.001
No	1 [Reference]	NA	1 [Reference]	NA
Read to child ≥4 d/wk				
Yes	0.01 (0.01)	.17	0.03 (0.01)	.002
No	1 [Reference]	NA	1 [Reference]	NA
Tell stories or sing songs to child ≥4 d/wk				
Yes	0.10 (0.01)	<.001	0.05 (0.01)	<.001
No	1 [Reference]	NA	1 [Reference]	NA
Family meal ≥4 d/wk				
Yes	0.12 (0.01)	<.001	0.11 (0.01)	<.001
No	1 [Reference]	NA	1 [Reference]	NA
Study group				
Second-generation Asian American	−0.006 (0.02)	.71	0.01 (0.02)	.68
Third- or later-generation Asian American	0.03 (0.03)	.44	−0.03 (0.03)	.34
Third- or later-generation White American	1 [Reference]	NA	1 [Reference]	NA

Abbreviation: NA, not applicable.

^a^ All characteristics were self-reported. A higher score indicates better social-emotional development and higher self-regulation.

Survey year–specific analyses showed consistent results with 5-year combined analyses (eTables 1-3 in Supplement 1). Subgroup analyses that included the second- and third- or later-generation Asian American groups showed that regular reading and regular family meals were positively associated with regular bedtimes and moderate screen time, and regular storytelling or singing was positively associated with flourishing (eTable 4 in Supplement 1). Analysis results with imputed data are presented in eTables 5 and 6 in Supplement 1.

## Discussion

This study found that Asian American children were less likely to flourish, have regular bedtimes, and have moderate screen time compared with White children in a US national sample. Among Asian American children, second-generation children were less likely to flourish compared with third- or later-generation children. This study further suggests that compared with US-born Asian parents, Asian immigrant parents were less likely to engage in positive parenting practices of regular reading and storytelling or singing, which were positively associated with flourishing, regular bedtimes, and moderate screen time. Lower likelihoods of flourishing, regular bedtimes, and moderate screen time among Asian American children remained significant after accounting for positive parenting practices. Among children aged 3 to 5 years, positive parenting practices were associated with better social-emotional development and self-regulation. However, no significant differences were found in social-emotional development and self-regulation among second-generation Asian American, third- or later-generation Asian American, and third-generation or later White children.

### Social and Familial Context and Acculturation Among Asian American Children by Generational Status

Generational status is often used to indicate acculturation level among Asian American populations.^30,31^ The current study found different social and familial context by generational status among Asian American families, consistent with prior generation studies.^32,33^ More than half of the families of second-generation children did not speak English at home, whereas this was true for only 6.3% of families of third- or later-generation children. Parents of second-generation Asian American children had a lower proportion of emotional support for parenting compared with parents of third- or later-generation Asian American children. Among Asian immigrant parents, language barriers and lack of emotional support for parenting may partly explain less frequent engagement in positive parenting practices and their offspring’s reduced psychological well-being.

This study is significant in that it is one of the first investigations of the status of a few psychological and behavioral variables in a large national sample of Asian American children using the existing data. However, the data sources lack information about cultural, social, and familial contexts of second-generation and third-generation Asian American children. In particular, without the country of origin (Asian subgroups) or cultural context data of participants, cultural implications cannot be fully explored because Asian subgroups present diverse cultural and immigrant backgrounds.

### Psychological Well-Being in Young Asian American Children

A prior study using 2018 to 2021 NSCH data^28^ found that Asian American children aged 0 to 5 years were less likely to flourish compared with their White counterparts. The current study expanded on these prior findings and revealed that second-generation Asian American children, compared with third- or later-generation Asian American children, were less likely to flourish, while there were no meaningful generational differences in self-regulation or social-emotional scores. Among the 4 flourishing items, resilience showed the largest differences across the study groups. This finding may indicate some potential difficulties among Asian American children with bouncing back quickly from setbacks. Lower emotional support for parenting among Asian immigrant parents may negatively influence their offspring’s psychological well-being. However, these findings should be interpreted with caution because the flourishing measure was a parent-reported subjective measure developed based on norms within the dominant Western culture. Beliefs and values derived from Asian cultures, such as an emphasis on emotional self-control,^34^ may establish different parental expectations for children in relation to bouncing back quickly from setbacks (eg, expecting a quicker recovery from setbacks). Therefore, although these findings underscore potentially lower flourishing among young Asian American children, it is crucial to further explore the extent to which these differences are due to nuanced cultural perceptions by validating the flourishing measure among Asian American families. We recommend that future investigations validate the measure and conduct an evaluation of flourishing among disaggregate Asian subgroups.

### Health Behaviors Among Young Asian American Children

A regular bedtime and moderate screen time are important childhood health behaviors. A regular bedtime is positively associated with children’s cognitive development and behavioral self-regulation.^26,35^ Although most young Asian American children (>90%) in this study had a regular bedtime, the likelihood of having a regular bedtime was slightly lower among Asian American children compared with White children (94.3%). These findings could be related to lack of a bedtime routine; Mindell et al^36^ reported that children from Asian countries were less likely to have a regular bedtime routine compared with those from predominantly Western countries, demonstrating cross-cultural differences.

Excessive screen time has been shown to negatively impact children’s cognitive, language, and social-emotional development.^37,38,39,40,41,42,43,44,45^ Although there were no differences by generational status, we observed a lower likelihood of moderate screen time among Asian American children compared with White children, consistent with a prior study.^46^ Although this finding should be replicated using an objective assessment of screen time, it highlights a potentially elevated risk of excessive screen time in young Asian American child populations.

### Associations of Parenting Practices With Children’s Psychological Well-Being and Health Behaviors Among Asian American Families

Parents’ participation in and parent-child interactions during literacy activities, such as reading and storytelling, are foundational to children’s language growth and cognitive development.^47,48^ Similarly, family meals have been shown to positively impact children’s social-emotional and behavioral development.^48,49^ Consistent with this knowledge, we found that engagement in positive parenting practices of regular reading, storytelling or singing, and family meals was associated with better psychological well-being and health behaviors among young Asian American children. Although parenting values and practices vary across cultures,^50^ these findings confirm that the positive parenting practices measured here, which were identified and developed based on norms within a dominant Western culture, are also significant to early childhood development and health in Asian American populations.

### Parenting Practices Among Asian American Families

Our findings of a lower likelihood of regular engagement in positive parenting practices among families of second-generation US children are consistent with a prior study^51^ showing that racial and ethnic minority children from non–English-speaking households had fewer parent-child interactions involving reading and storytelling compared with children without these characteristics. These findings could partly reflect a lack of emphasis on healthy daily routines (ie, bedtime or dinnertime routines) that involve reading, storytelling or singing, and family meals among families of second-generation US children. Due to its association with an array of positive developmental outcomes, a healthy bedtime routine (ie, inclusive of adaptive activities such as reading and singing, and less screen time) has been suggested as a feasible and cost-effective strategy to promote positive early childhood development worldwide.^52^ Thus, promotion of a healthy bedtime routine is recommended to improve health behaviors as well as psychological well-being outcomes among Asian American children.

Our finding of a lower likelihood of a regular engagement in positive parenting practices for second-generation Asian American children than for third- or later-generation Asian American children highlights the importance of considering generational status when designing programs and policies. Although generational status is not amenable to interventions, programs to educate Asian immigrant parents about positive parenting practices could help reduce the gap in these practices across Asian American generations and racial and ethnic groups. However, such programming will require a deeper understanding of barriers to reading and storytelling in these families. For example, Asian immigrant parents may have limited access to children’s books in their primary language or face language barriers. Additionally, continued investigation of young children’s daily routines (eg, bedtime or mealtime routines) among Asian American families will be important for designing effective interventions.

### Limitations

This study has some limitations. First, a major limitation of this study was an inability to disaggregate data by Asian ethnic subgroups due to a lack of subgroup information in the NSCH datasets. The psychological well-being measures used in this study were not validated among Asian American young children, which is another limitation of this study. As previous research suggests that mental health status can differ substantially across Asian subgroups,^53^ our current findings may not directly apply to certain Asian subpopulations. Second, findings from this cross-sectional investigation cannot be used to establish a temporal relationship between parenting practices and the child outcomes investigated. Furthermore, proxy-reported measures for psychological well-being and health behaviors could have caused measurement error.^54^ Third, the low annual response rate that ranged from 37% to 42% is a limitation. Fourth, unmeasured confounding factors may have biased study results.

## Conclusions

Our study showed lower likelihoods of flourishing, regular bedtimes, and moderate screen time among Asian American children compared with non-Hispanic White children in the US. Reading and storytelling or singing parenting practices, which were associated with children’s well-being and health behaviors, were particularly less frequent among parents of second-generation Asian American children than those of third- or later-generation Asian American children. Promotion of these practices among Asian immigrant parents may help improve the psychological well-being and health behaviors of their young Asian American children.

## Notes

Supplement 1. **eTable 1.** Adjusted Odds Ratios for the Flourishing Outcome by Survey Years Among 2018-2022 NSCH Participants Aged 0-5 Years **eTable 2.** Adjusted Odds Ratios for Regular Bedtimes by Survey Years Among 2018-2022 NSCH Participants Aged 0-5 Years **eTable 3.** Adjusted Odds Ratios for Moderate Screen Time by Survey Years Among 2018-2022 NSCH Participants Aged 0-5 Years **eTable 4.** Adjusted Odds Ratios for Flourishing and Health Behavior Outcomes Among 2018-2022 NSCH Asian Participants Aged 0-5 Years **eTable 5.** Multivariable Logistic Regression Models for Flourishing, Regular Bedtime, and Moderate Screen Time Among 2018-2022 NSCH Participants Aged 0-5 Years, Using the Imputed Data for Mother’s Education and Neighborhood Support **eTable 6.** Multivariable Linear Regression Models for Social-Emotional and Self-Regulation Scores Among 2018-2022 NSCH Participants Aged 3-5 Years, Using the Imputed Data for Mother’s Education and Neighborhood Support

Supplement 2.
Data Sharing Statement
