# Supplementary material for: Parenting Practices and Well-Being and Health Behaviors Among Young Asian American Children
Source: JAMA Netw Open. 2025 Jan 13;8(1):e2454516. doi: 10.1001/jamanetworkopen.2024.54516 (PMC11731191; doi:10.1001/jamanetworkopen.2024.54516)
Supplement: Supplement 1. — eTable 1. Adjusted Odds Ratios for the Flourishing Outcome by Survey Years Among 2018-2022 NSCH Participants Aged 0-5 Years eTable 2. Adjusted Odds Ratios for Regular Bedtimes by Survey Years Among 2018-2022 NSCH Participants Aged 0-5 Years eTable 3. Adjusted Odds Ratios for Moderate Screen Time by Survey Years Among 2018-2022 NSCH Participants Aged 0-5 Years eTable 4. Adjusted Odds Ratios for Flourishing and Health Behavior Outcomes Among 2018-2022 NSCH Asian Participants Aged 0-5 Years eTable 5. Multivariable Logistic Regression Models for Flourishing, Regular Bedtime, and Moderate Screen Time Among 2018-2022 NSCH Participants Aged 0-5 Years, Using the Imputed Data for Mother’s Education and Neighborhood Support eTable 6. Multivariable Linear Regression Models for Social-Emotional and Self-Regulation Scores Among 2018-2022 NSCH Participants Aged 3-5 Years, Using the Imputed Data for Mother’s Education and Neighborhood Support [file jamanetwopen-e2454516-s001.pdf]

Supplementary Online Content

Kwon S, Shin ED, Bartell TR, Capan S. Psychological well-being and behaviors among young Asian American children. *JAMA Netw Open*. 2025;8(1):e2454516. doi:10.1001/jamanetworkopen.2024.54516

eTable 1. Adjusted Odds Ratios for the Flourishing Outcome by Survey Years Among 2018-2022 NSCH Participants Aged 0-5 Years
eTable 2. Adjusted Odds Ratios for Regular Bedtimes by Survey Years Among 2018-2022 NSCH Participants Aged 0-5 Years
eTable 3. Adjusted Odds Ratios for Moderate Screen Time by Survey Years Among 2018-2022 NSCH Participants Aged 0-5 Years
eTable 4. Adjusted Odds Ratios for Flourishing and Health Behavior Outcomes Among 2018-2022 NSCH Asian Participants Aged 0-5 Years
eTable 5. Multivariable Logistic Regression Models for Flourishing, Regular Bedtime, and Moderate Screen Time Among 2018-2022 NSCH Participants Aged 0-5 Years, Using the Imputed Data for Mother's Education and Neighborhood Support
eTable 6. Multivariable Linear Regression Models for Social-Emotional and Self-Regulation Scores Among 2018-2022 NSCH Participants Aged 3-5 Years, Using the Imputed Data for Mother's Education and Neighborhood Support

This supplementary material has been provided by the authors to give readers additional information about their work.

© 2025 Kwon S et al. *JAMA Network Open.*

eTable 1. Adjusted Odds Ratios for the Flourishing Outcome by Survey Years Among 2018-2022 NSCH Participants Aged 0-5 Years

	2018-2019	2020	2021	2022
	OR (95% CI)	OR (95% CI)	OR (95% CI)	OR (95% CI)
Reading to child ≥4 d/wk: yes vs. no	1.29 (1.11, 1.50)	1.20 (1.01, 1.42)	1.11 (0.98, 1.26)	1.05 (0.93, 1.20)
Tell stories or sing songs to child ≥4 d/wk: yes vs. no	1.19 (1.12, 1.39)	1.34 (1.12, 1.59)	1.44 (1.27, 1.63)	1.41 (1.25, 1.60)
Family meal ≥4 d/wk: yes vs. no	1.10 (0.93, 1.30)	1.32 (1.09, 1.60)	1.36 (1.18, 1.56)	1.27 (1.10, 1.45)
Study group: 2nd generation Asian vs. 3rd or later generation White	0.42 (0.33, 0.52)	0.60 (0.47, 0.77)	0.61 (0.51, 0.73)	0.66 (0.55, 0.79)
Study group: 3rd or later generation Asian vs. 3rd or later generation White	0.86 (0.46, 1.59)	0.79 (0.46, 1.36)	0.92 (0.61, 1.38)	0.79 (0.53, 1.16)

Adjusted for age, sex, federal poverty level, respondent’s marital status, respondent having emotional support for parenting, maternal education, and neighborhood support.
CI, confidence interval; OR, odds ratio.

© 2025 Kwon S et al. *JAMA Network Open.*

eTable 2. Adjusted Odds Ratios for Regular Bedtimes by Survey Years Among 2018-2022 NSCH Participants Aged 0-5 Years

	2018-2019	2020	2021	2022
	OR (95% CI)	OR (95% CI)	OR (95% CI)	OR (95% CI)
Reading to child ≥4 d/wk: yes vs. no	2.02 (1.68, 2.43)	1.96 (1.56, 2.47)	2.40 (2.00, 2.87)	1.97 (1.64, 2.37)
Tell stories or sing songs to child ≥4 d/wk: yes vs. no	1.15 (0.95, 1.39)	1.52 (1.21, 1.90)	1.17 (0.97, 1.40)	1.16 (0.96, 1.40)
Family meal ≥4 d/wk: yes vs. no	1.43 (1.18, 1.74)	1.56 (1.22, 1.98)	1.49 (1.23, 1.80)	1.73 (1.44, 2.08)
Study group: 2nd generation Asian vs. 3rd or later generation White	0.64 (0.47, 0.87)	0.94 (0.65, 1.36)	0.98 (0.75, 1.30)	0.70 (0.54, 0.91)
Study group: 3rd and later generation Asian vs. 3rd or later generation White	0.91 (0.41, 2.00)	0.60 (0.30, 1.21)	0.52 (0.33, 0.84)	0.76 (0.43, 1.34)

Adjusted for age, sex, federal poverty level, respondent’s marital status, respondent having emotional support for parenting, maternal education, and neighborhood support.
CI, confidence interval; OR, odds ratio.

eTable 3. Adjusted Odds Ratios for Moderate Screen Time by Survey Years Among 2018-2022 NSCH Participants Aged 0-5 Years

	2018-2019	2020	2021	2022
	OR (95% CI)	OR (95% CI)	OR (95% CI)	OR (95% CI)
Reading to child ≥4 d/wk: yes vs. no	1.31 (1.19, 1.45)	1.23 (1.08, 1.39)	1.32 (1.20, 1.45)	1.49 (1.35, 1.65)
Tell stories or sing songs to child ≥4 d/wk: yes vs. no	1.03 (0.93, 1.14)	1.06 (0.93, 1.21)	1.13 (1.03, 1.25)	0.97 (0.87, 1.07)
Family meals ≥4 d/wk: yes vs. no	1.61 (1.44, 1.79)	1.74 (1.50, 2.03)	1.45 (1.30, 1.61)	1.56 (1.40, 1.74)
Study group: 2nd generation Asian vs. 3rd or later generation White	0.82 (0.68, 0.99)	0.75 (0.61, 0.93)	0.95 (0.81, 1.10)	1.07 (0.92, 1.25)
Study group: 3rd or later generation Asian vs. 3rd or later generation White	1.01 (0.66, 1.54)	0.67 (0.44, 1.02)	0.68 (0.51, 0.92)	1.02 (0.74, 1.40)

Moderate screen time was defined as less than 1 hour/day for children aged 0-1 and 1 hour/day or less for ages 2-5. Adjusted for age, sex, federal poverty level, respondent's marital status, respondent having emotional support for parenting, maternal education, and neighborhood support.
CI, confidence interval; OR, odds ratio.

© 2025 Kwon S et al. *JAMA Network Open.*

eTable 4. Adjusted Odds Ratios for Flourishing and Health Behavior Outcomes Among 2018-2022 NSCH Asian Participants Aged 0-5 Years

	Flourishing	Regular bedtime	Moderate screen time[a]
	OR (95% CI)	OR (95% CI)	OR (95% CI)
Reading to child ≥4 d/wk: yes vs. no	1.11 (0.91, 1.34)	1.84 (1.38, 2.46)	1.53 (1.29, 1.82)
Tell stories or sing songs to child ≥4 d/wk: yes vs. no	1.34 (1.09, 1.63)	0.99 (0.75, 1.32)	1.11 (0.93, 1.32)
Family meal together ≥4 d/wk: yes vs. no	0.96 (0.77, 1.18)	1.33 (1.01, 1.76)	1.39 (1.15, 1.67)
2nd generation vs. 3rd or later generation Asian	0.72 (0.56, 0.93)	1.17 (0.85, 1.63)	1.12 (0.92, 1.36)

[a]Moderate screen time was defined as less than 1 hour/day for children aged 0-1 and 1 hour/day or less for ages 2-5.

Adjusted for age, sex, federal poverty level, respondent's marital status, respondent having emotional support for parenting, maternal education, and neighborhood support.

CI, confidence interval; OR, odds ratio.

eTable 5. Multivariable Logistic Regression Models for Flourishing, Regular Bedtime, and Moderate Screen Time Among 2018-2022 NSCH Participants Aged 0-5 Years, Using the Imputed Data for Mother's Education and Neighborhood Support

	Flourishing (n=42316)		Regular bedtime (n=42568)		Moderate screen time (n=42530)	
	OR	95% CI	OR	95% CI	OR	95% CI
Age, years	0.83	0.82, 0.85	0.96	0.94, 0.99	0.77	0.76, 0.78
Sex						
Male	0.75	0.78, 0.80	1.11	1.02, 1.20	0.97	0.93, 1.01
Female	Ref	Ref	Ref	Ref	Ref	Ref
Federal poverty level						
<100% (below poverty level)	0.56	0.50, 0.64	0.57	0.49, 0.67	0.88	0.80, 0.97
100 to <200%	0.77	0.70, 0.85	0.75	0.66, 0.86	0.85	0.79, 0.91
200 to <400%	0.86	0.81, 0.93	0.85	0.77, 0.95	0.83	0.79, 0.87
≥400%	Ref	Ref	Ref	Ref	Ref	Ref
Respondent's marital status						
Non-married	0.97	0.89, 1.06	0.66	0.59, 0.74	0.93	0.87, 1.00
Married	Ref	Ref	Ref	Ref	Ref	Ref
Respondent has emotional support for parenting						
Yes	1.51	1.39, 1.65	1.42	1.26, 1.60	1.09	1.02, 1.17
No	Ref	Ref	Ref	Ref	Ref	Ref
Mother's education						
<Bachelor's degree	0.94	0.88, 1.01	0.75	0.68, 0.83	0.60	0.57, 0.63
≥Bachelor's degree	Ref	Ref	Ref	Ref	Ref	Ref
Neighborhood support						
Yes	1.57	1.48, 1.67	1.38	1.27, 1.50	1.27	1.22, 1.33
No	Ref	Ref	Ref	Ref	Ref	Ref
Read to child ≥4 days/week						
Yes	1.15	1.07, 1.23	2.12	1.92, 2.33	1.33	1.27, 1.40
No	Ref	Ref	Ref	Ref	Ref	Ref
Tell stories or sing songs to child ≥4 days/week						
Yes	1.36	1.27, 1.47	1.25	1.13, 1.38	1.05	1.00, 1.11
No	Ref	Ref	Ref	Ref	Ref	Ref
Family meal ≥4 days/week						
Yes	1.26	1.17, 1.36	1.55	1.40, 1.72	1.56	1.47, 1.66
No	Ref	Ref	Ref	Ref	Ref	Ref
Study group						
2nd generation Asian	0.58	0.52, 0.64	0.81	0.69, 0.94	0.91	0.84, 1.10
3rd or later generation Asian	0.80	0.63, 1.02	0.68	0.49, 0.93	0.81	0.67, 0.97
3rd or later generation White	Ref	Ref	Ref	Ref	Ref	Ref

All characteristics were self-reported.
CI, confidence interval; OR, odds ratio; Ref, reference.
*Multiple imputation by chained equations (MICE) method was used for imputation of missing data for the mother's education and neighborhood support variables.

© 2025 Kwon S et al. *JAMA Network Open.*

eTable 6. Multivariable Linear Regression Models for Social-Emotional and Self-Regulation Scores Among 2018-2022 NSCH Participants Aged 3-5 Years, Using the Imputed Data for Mother's Education and Neighborhood Support

	Social-emotional score (n=23785)		Self-regulation score (n=23767)	
	Coefficient±SE	P-value	Coefficient±SE	P-value
Intercept	3.46±0.03	<0.001	3.28±0.03	<0.001
Age, years	0.12±0.00	<0.001	0.05±0.00	<0.001
Sex				
Male	-0.14±0.01	<0.001	-0.12±0.01	<0.001
Female	Ref	Ref	Ref	Ref
Federal poverty level				
<100% (below federal poverty level)	-0.13±0.02	<0.001	-0.11±0.02	<0.001
100 to <200%	-0.06±0.01	<0.001	-0.03±0.01	0.03
200 to <400%	-0.03±0.01	0.002	-0.01±0.01	0.32
≥400%	Ref	Ref	Ref	Ref
Respondent's marital status				
Non-married	0.02±0.01	0.20	-0.07±0.01	<0.001
Married	Ref	Ref	Ref	Ref
Respondent has emotional support for parenting				
Yes	0.10±0.01	<0.001	0.13±.01	<0.001
No	Ref	Ref	Ref	Ref
Mother's education				
<Bachelor's degree	0.05±0.01	<0.001	-0.03±0.01	0.004
≥Bachelor's degree	Ref	Ref	Ref	Ref
Neighborhood support				
Yes	0.18±0.01	<0.001	0.15±0.01	<0.001
No	Ref	Ref	Ref	Ref
Read to child ≥4 days/week				
Yes	0.01±0.01	0.14	0.03±0.01	0.002
No	Ref	Ref	Ref	Ref
Tell stories or sing songs to child ≥4 days/week				
Yes	0.11±0.01	<0.001	0.05±0.01	<0.001
No	Ref	Ref	Ref	Ref
Family meal ≥4 days/week				
Yes	0.12±0.01	<0.001	0.11±0.01	<0.001
No	Ref	Ref	Ref	Ref
Study group				
2nd generation Asian	0.01±0.02	0.75	0.02±0.02	0.26
3rd or later generation Asian	0.02±0.03	0.66	-0.04±0.03	0.22
3rd or later generation White	Ref	Ref	Ref	Ref

All characteristics were self-reported.
A higher score indicates better social-emotional development and higher self-regulation.
Ref, reference; SE, standard error.
*Multiple imputation by chained equations (MICE) method was used for imputation of missing data for the mother's education and neighborhood support variables.

© 2025 Kwon S et al. *JAMA Network Open.*
