# Supplementary material for: Parenting Practices and Well-Being and Health Behaviors Among Young Asian American Children
Source: JAMA Netw Open. 2025 Jan 13;8(1):e2454516. doi: 10.1001/jamanetworkopen.2024.54516 (PMC11731191; doi:10.1001/jamanetworkopen.2024.54516)
Supplement: Supplement 2. — Data Sharing Statement [file jamanetwopen-e2454516-s002.pdf]

Data Sharing Statement

Kwon. Psychological Well-Being and Behaviors Among Young Asian American Children. *JAMA Netw Open*. Published January 13, 2025. doi:10.1001/jamanetworkopen.2024.54516

Data
Data available: Yes
Data types: Deidentified participant data, Data dictionary, Other (please specify)
Additional Information: Publicly available data/data dictionary
How to access data: https://www.childhealthdata.org/learn-about-the-nsch/NSCH
When available: With publication

Supporting Documents
Document types: None

Additional Information
Who can access the data: Data are publicly available.
Types of analyses: Any purpose.
Mechanisms of data availability: Data are publicly available.
